# Infant Fecal Fermentations with Galacto-Oligosaccharides and 2′-Fucosyllactose Show Differential *Bifidobacterium longum* Stimulation at Subspecies Level

**DOI:** 10.3390/children10030430

**Published:** 2023-02-23

**Authors:** Cordula Lindner, Ellen Looijesteijn, Helmie van Dijck, Ingeborg Bovee-Oudenhoven, Margreet Heerikhuisen, Tim J. van den Broek, Massimo Marzorati, Vassilis Triantis, Arjen Nauta

**Affiliations:** 1 FrieslandCampina, 3818 LA Amersfoort, The Netherlands; 2 The Netherlands Organization for Applied Scientific Research (TNO), Microbiology and Systems Biology, 2333 BE Leiden, The Netherlands; 3 ProDigest, 9052 Ghent, Belgium; 4 Center for Microbial Ecology and Technology, Faculty of Bioscience Engineering, University of Ghent, 9000 Ghent, Belgium

**Keywords:** galacto-oligosaccharides, 2′-fucosyllactose, fecal microbiota, bifidobacterial, infants, toddlers, short chain fatty acids

## Abstract

The objective of the current study was to evaluate the potential of 2′-FL and GOS, individually and combined, in beneficially modulating the microbial composition of infant and toddler (12–18 months) feces using the micro-Matrix bioreactor. In addition, the impacts of GOS and 2′-FL, individually and combined, on the outgrowth of fecal bifidobacteria at (sub)species level was investigated using the baby M-SHIME^®^ model. For young toddlers, significant increases in the genera *Bifidobacterium*, *Veillonella*, and *Streptococcus*, and decreases in *Enterobacteriaceae*, *Clostridium* XIVa, and *Roseburia* were observed in all supplemented fermentations. In addition, GOS, and combinations of GOS and 2′-FL, increased *Collinsella* and decreased *Salmonella*, whereas 2′-FL, and combined GOS and 2′-FL, decreased *Dorea*. Alpha diversity increased significantly in infants with GOS and/or 2′-FL, as well as the relative abundances of the genera *Veillonella* and *Akkermansia* with 2′-FL, and *Lactobacillus* with GOS. Combinations of GOS and 2′-FL significantly stimulated *Veillonella*, *Lactobacillus*, *Bifidobacterium*, and *Streptococcus*. In all supplemented fermentations, Proteobacteria decreased, with the most profound decreases accomplished by the combination of GOS and 2′-FL. When zooming in on the different (sub)species of *Bifidobacterium*, GOS and 2’-FL were shown to be complementary in stimulating breast-fed infant-associated subspecies of *Bifidobacterium longum* in a dose-dependent manner: GOS stimulated *Bifidobacterium longum* subsp. *longum*, whereas 2′-FL supported outgrowth of *Bifidobacterium longum* subsp. *infantis*.

## 1. Introduction

Early microbiota colonization and development is important for immune development and health status throughout life [1,2,3,4,5,6]. A natural, gradual, and healthy colonization and microbiota development in the gut is greatly stimulated by a term and vaginal birth, and subsequent breast-feeding [7,8,9,10,11,12,13]. High relative proportions of bifidobacteria in the gut are believed to be beneficial due to, amongst others, their high capacity for producing short chain fatty acids (SCFA), in particular acetate. This lowers the intestinal pH and creates an environment favoring acid tolerant bifidobacteria and lactobacilli and disfavoring outgrowths of Proteobacteria like *Escherichia coli* [14,15,16]. Other important benefits of bifidobacteria are their ability to produce antimicrobial peptides and vitamins, their anti-oxidant and immune-modulatory properties, their role in mucosal immunity, and their ability to produce metabolites that mediate gut–brain communication such as indole-3-lactate [3,17,18,19,20,21,22,23].

Human milk is tuned to nourish and protect the infant optimally, but also to shape the developing gut microbiota. Formula-fed infants have been shown to possess lower levels of the genus *Bifidobacterium* as compared to breast-fed infants [24,25,26,27]. Human milk oligosaccharides (hMOs) play a primary role in this generic bifidogenic effect [28,29,30,31,32,33]. To compensate for the lack of hMOs in basic infant formula and grown-up milk (IF, GUM), galacto-oligosaccharides (GOS) and other prebiotic oligosaccharides have been added, for their microbiota supporting properties. The bifidogenicity of GOS is well substantiated in different in vitro settings, with single strains [34,35,36] and fecal cultures of infants [37,38], as well as in vivo in infants [39,40,41,42,43,44]. Since the introduction of prebiotic oligosaccharides in IF, the obvious initial difference between *Bifidobacterium* levels in the stools of breast- and formula-fed infants has become much smaller. Today, formula-fed infants harbor equal or even higher portions of the genus *Bifidobacterium* [45,46,47,48]. For 5–10-year-old children [49], adults [50,51,52,53], and the elderly [54], bifidogenic effects have been demonstrated for GOS. Although GOS, in combination with inulin and either fructo-oligosaccharides or *Bifidobacterium breve* have been shown to promote the outgrowth of bifidobacteria in vivo in toddlers of 1–3 years [55,56], as far as we know, the effects of solely GOS on the toddler fecal microbiome have not been reported.

In most populations, around 80% of people are secretor positive, meaning that they possess a functional fucosyltransferase 2 gene, to produce 2′-fucosylated oligosaccharides [57]. The breast milk of secretor positive mothers contains high levels of the neutral hMO, 2′-fucosyllactose (2′-FL), which is absent in human milk of secretor negative mothers [58,59]. As the first hMO that has been applied in IF, 2′-FL is increasingly linked with benefits related to infants’ health. 2′-FL is especially known for its immune modulating and anti-infection properties [60], which have been shown in models for necrotizing enterocolitis [61,62], vaccination [63], and enteric or respiratory infections [64,65,66]. Clinical studies showed that 2′-FL might support the immune and gut health of infants, gives rise to fewer respiratory infections, and reduces formula intolerance [67,68,69].

Shifts of fecal bacteria and metabolic profiles to being more similar to those of breast-fed infants, have been reported for the combination of 2′-FL with lacto-N-neotetraose, another neutral hMO [70]. A bifidogenic effect of 2′-FL has been proposed, based on observational association studies comparing the fecal microbiota composition of infants of either secretor positive or secretor negative mothers [71,72,73]. The ability of 2′-FL to support the growth of bifidobacteria has been shown in a number of in vitro studies with selected bifidobacterial strains and fecal samples, or indirectly by the consumption of 2′-FL [74,75,76,77,78,79,80,81,82,83,84,85,86]. Recently, it was further demonstrated that 2′-FL increases the rate of GOS fermentation in an in vitro fermentation with infant feces. In this fermentation, the generic bifidogenic effect of combined GOS and 2′-FL was somewhat higher than that of the single components [85]. To the best of our knowledge, a direct comparison of the modulating effects of 2′-FL, GOS, and combinations thereof, on the gut microbiota of toddlers, has not been described before.

The aim of the current study was to evaluate the potential of 2′-FL and GOS, individually and combined, in modulating the microbiota composition of infants’ and toddlers’ (12–18 months) feces, using a pH-controlled batch culture model. In addition, the impact of GOS and 2′-FL in infants, individually and combined, on the outgrowth of fecal bifidobacteria at (sub)species level was revealed, using the dynamic baby M-SHIME^®^ model.

## 2. Materials and Methods

### 2.1. In Vitro Fecal Fermentation Models

Two different in vitro fecal fermentation models were employed in this study: the micro-Matrix bioreactor and the baby M-SHIME^®^ model.

Micro-Matrix bioreactor: Fecal batch fermentations were performed using the micro-Matrix (Applikon Biotechnology, Delft, The Netherlands), a mini fermentation batch culture model (1–10 mL) with pH control [87], simulating the proximal colon.

Baby M-SHIME^®^ model: The baby M-SHIME^®^ (Simulator of the Human Intestinal Microbial Ecosystem, ProDigest, Ghent, Belgium) is a dynamic fermentation model consisting of five reactors, simulating the various parts of the human gastrointestinal tract: stomach (St), small intestine (SI), ascending (AC), transverse (TC), and descending (DC) colon [88,89]. Procedures related to preparation of inoculum, settings of pH, temperature, and retention time, and medium composition, were reported before [89]. The M-SHIME^®^ constitutes a further development of the model, and includes the mucus compartment [90,91]. Moreover, the model was adapted to infant conditions with respect to retention (transit) times and pH [92].

### 2.2. Fecal Donors and Inoculum Preparation

#### 2.2.1. Micro-Matrix Bioreactor

For the micro-Matrix bioreactor fecal batch fermentations, two pools of either infant or toddler fecal samples were used. Each pool consisted of 10 individual fecal samples from healthy Caucasian donors of both genders. The infant donors were between one and six months of age, the toddler donors were between 12 and 18 months old. Exclusion criteria were, being ill (as judged by the parents), having fever or diarrhea preceding or during donation, antibiotic use during the last four weeks before feces collection and for infants also during the perinatal period. In addition, the infants were naturally born at term and did not receive breast milk in the four weeks prior to sampling. Although there is no information available about the type of IF fed to the infants, most of the formulas likely contained prebiotics. The parents of the donors gave written informed consent. All feces collected from diapers were kept in an anaerobic environment in a closed box (jar with Anaerocult A, MERCK, Darmstadt, Germany, following The Netherlands Organisation for Applied Scientific Research (TNO, The Netherlands) standard operational procedures [93]). These procedures are aimed at preserving the original microbial composition and activity as much as possible. The jars were opened inside an anaerobic cabinet (Bactron600, Sheldon Manufacturing, USA), with an atmospheric composition consisting of 80% N_2_, 10% H_2_, and 10% CO_2_, and operated according to the instructions of the manufacturer. Anaerobicity inside the anaerobic cabinet was verified using an indicator fluid (Anaerobic Indicator Solution B, Don Whitley, UK). The feces were processed in the anaerobic cabinet by mixing individual feces samples with phosphate-buffered saline (0.05 M, pH 7.2) containing 0.05% cysteine-HCl solution and glycerol (final concentration 20%) to a fecal slurry (20% *w*/*v*), homogenized, aliquoted, snap-frozen in liquid nitrogen, and kept at −80 °C. The maximum allowed time between collection of the fecal samples and storage of the fecal slurries at −80 °C was 8 h. Just before inoculation, individual fecal slurries were thawed and pooled in a standardized way, to guarantee that the microbiota composition at the start of the three triplicate experiments was similar.

#### 2.2.2. Baby M-SHIME^®^ Model

The fecal pool used for the baby M-SHIME^®^ experiments was composed of four donor feces from infants between 1.5 to 2 months of age, with the same exclusion criteria as for the micro-Matrix bioreactor fermentations, but in addition it was required that they did not receive any pre- or probiotics in the month before sampling. Fecal samples were collected from diapers in a similar way as described for the feces collected for the micro-Matrix bioreactor fermentations.

### 2.3. Media and Substrates

#### 2.3.1. Medium for Micro-Matrix Bioreactor

Standard carbohydrate-poor fermentation medium [94] was used in the micro-Matrix bioreactor batch culture fermentations. It contains the following components (g/L): peptone (2), 2 yeast extract (2), K_2_HPO_4_.3H_2_O (0.05), KH_2_PO_4_ (0.04), NaHCO_3_ (2), NaCl (0.1), MgSO_4_.7H_2_O (0.01), cysteine-HCl (0.5), hemin (0.005), bile (0.5), Tween 80 (2), vitamin K1 (0.01), and 0.025% Resazurin-solution (4), with the pH adjusted to 6.8.

#### 2.3.2. Medium for Baby M-SHIME^®^ Model

Based on the earlier developed baby SHIME medium [92], the baby M-SHIME^®^ medium used in this study was modified to better match the characteristics of infant formula-fed babies. Instead of 30 g/L undigested infant formula, 0.2 g/L casein, 2.7 g/L whey protein (lactalbumin), and 2.1 g/L lactose were added. These amounts were calculated based on previously published data [95]. Furthermore, the mucin content was reduced by 50%, because mucin was also added via the mucin (type II, Sigma) coated microcosms immersed into the vessels to simulate the mucus compartment of the colon.

#### 2.3.3. Substrates

The oligosaccharides studied were nanofiltration purified GOS [96] and 2′-FL (FrieslandCampina Ingredients, Amersfoort, The Netherlands).

### 2.4. Experimental Setup

#### 2.4.1. Micro-Matrix Bioreactor

The micro-Matrix bioreactor allows 24 small fermentations to be run in parallel by using the 24 wells micro-Matrix cassette. The conditions applied were: medium control, and oligosaccharide treatments in two different dosages (2.5 g/L and 10 g/L) with, GOS, 2′-FL, or GOS and 2′-FL in two different ratios, 1:1 (mix 1) and 3:1 (mix 2). Mix 2 was only studied at a dose of 10 g/L. All conditions were studied with infant as well as toddler fecal material. Six replicates were run for each condition (two replicates of the same condition at the same time on three different days) to allow statistical analysis to be performed on the analyzed parameters.

Each (small bioreactor) well (4 mL working volume) was filled in the order, fermentation broth, oligosaccharide(s), fecal slurry. Prior to adding the fecal pool, the cassette with medium and oligosaccharides was put on the micro-Matrix for about 20 min to reduce the oxygen concentration to a value below 0.35 mg/L, and to warm the medium with oligosaccharides to 37 °C. Subsequently, the system was programmed to raise the pH of the medium to pH 6.8, using ammonium gas, and once this pH was reached, the plates were removed from the system and inoculated with the respective fecal pool in the anaerobic cabinet. After inoculation the reactor plate was incubated anaerobically for 24 h at 37 °C. After initial acidification of the medium to pH 5.8, the pH set-point was set at 5.9, with a dead zone of 0.1 pH-units. A pH range between 5.8 and 6.0 resembles the pH in the infant proximal colon [97]. The pH was maintained at the set-point by ammonia and CO_2_ gas. Nitrogen gas was used to create an anaerobic atmosphere. The plates were orbitally shaken at 300 rpm.

Samples (1 mL each) were taken at 6 and 24 h of fermentation, under anaerobic conditions in the anaerobic cabinet, and centrifuged for 5 min at 12,500× *g*. Supernatants and pellets were separately frozen and kept at −20 °C. For comparison, samples taken after 6 h of fermentation were analyzed, as this timepoint was found to be representative of a period of high fermentation activity and differentiation in an earlier pilot experiment (data not shown).

#### 2.4.2. Baby M-SHIME^®^ Model

The standard adult setup of the SHIME^®^ model (Prodigest, Belgium [98]) was adapted to mimic infant conditions [92] yet, with some adaptations. Briefly, during all experiments, a simulation of the mucosal environment was included, according to [90], while the simulation of the colon was restricted to the proximal colon region (V = 300 mL; pH = 5.6–5.8), and the medium was adapted as stated above (see Section 2.3.2). During the dose–response experiment, the model was operated as a QUINTUPLE-SHIME^®^, five units in parallel, where each of the five units consisted of a stomach/small intestine and proximal colon.

The experimental design consisted of 3 stages: a startup, control, and treatment phase. (i) To initiate the two-week startup period, the colon reactors were inoculated with the fecal pool. During this phase, basic nutritional medium (2.3.2) was added to the reactors to support the maximum diversity of the microbiota of the inoculum. (ii) During the one-week control period, the basic nutritional medium (2.3.2) was further added. Samples taken from the different reactors during the control period were used to assess the baseline microbiota composition and metabolic activity. (iii) In the 2-week treatment period, oligosaccharides were supplemented to the basic diet, either 100% GOS, 100% 2′-FL, or 90/10, 75/25, or 50/50% of GOS/2′-FL, to a final concentration of 3 g/L. Samples (1 mL each) were taken three times per week from the different compartments, and centrifuged for 5 min at 12,500× *g*. Supernatants and pellets were separately frozen and kept at −20 °C.

### 2.5. DNA Extraction from Fecal Slurries

One ml of each fermentation sample was pelleted via centrifugation at 12,500× *g* for 5 min, and DNA was isolated using either the QIAamp DNA Stool Mini Kit, from Qiagen, combined with Quickgene DNA tissue kit S, from FujiFilm (micro-Matrix samples), or by the Fast-Prep24 instrument (MP-BIO, Germany) (baby M-SHIME^®^ samples). The latter was carried out as previously described [99]. DNA quality and quantity were determined using agarose gels (1% *w*/*v*) and by measuring the absorbance at 260 nm, as well as the ratio of absorbance at 260 nm and 280 nm. All values were in-between 1.81 and 1.99, indicating pure DNA.

### 2.6. Microbiota Analysis Micro-Matrix Bioreactor Fermentations

#### 2.6.1. 16S rRNA Gene Amplicon Sequencing

Fecal DNA isolates from fecal pools and samples of the micro-Matrix fermentations were analyzed by using 16S rRNA paired-end amplicon sequencing, conducted on the Illumina MiSeq platform (Illumina, Eindhoven, The Netherlands). One thousand pg of DNA was amplified for each sample, as described previously [100], using 30 PCR cycles. The V4 region was targeted using F515 and R806 primers [101]. The primers included Illumina adapters and a unique 8-nucleotide sample index sequence key [100]. The amplicon libraries were then pooled in equimolar amounts and purified using the QIAquick Gel Extraction Kit (QIAGEN). Amplicon quality and size were analyzed on a Fragment Analyzer (Advanced Analytical Technologies Inc., Heidelberg, Germany), and filtered and processed according to the TNO pipeline, yielding 4,833,046 reads with an average of 26,850 reads per sample. The metataxonomic analysis provided identification to the genus level.

#### 2.6.2. Bioinformatic Sequencing Processing and Analysis

Sequence preprocessing, analysis, and classification were performed using modules of the Mothur software platform [102]. Chimeric sequences were identified and removed using the ‘chimera.uchime’ command. Unique sequences were aligned using the ‘align.seqs’ command and the Mothur compatible bacterial SILVA SEED database (Release 119). Taxonomic classification was performed using the RDP-II Naïve Bayesian Classifier, using a 60% confidence threshold against the RDP Database (Release 11.1) for 16S rRNA. Diversity, including the Shannon diversity index and Tail statistics, was calculated.

#### 2.6.3. Statistical Analyses of Sequencing Data

All statistical analyses of sequencing data were performed using R, version 4.1.2 [103]. The sequencing data was filtered to include only those taxa that contributed to the first 97.5% of all counts in the data. This step was not applied to the data for determination of alpha-diversity.

PerMANOVA (permutational analysis of variance) [104] was used to perform group-wise comparisons of the samples. This method was used to compare treatment groups and to test the (null) hypothesis that the centroids (group average) and the dispersion (distance of samples from their group average) of different groups were not significantly different from each other. The DESeq2 method [105] was applied to test the significance of the differences between the abundances of treatment and control groups for the bacterial taxa. This method performs a Wald test on a negative binomial generalized linear model for each observed microbial taxon (genus in this case), resulting in log2 fold changes between groups. Correction for multiple testing was performed using the Benjamini–Hochberg method, resulting in adjusted *p*-values. Statistical significance was accepted at *p* < 0.05.

The figures were generated using the ggplot2 package, version 3.3.5 [106].

Note that the two different mixtures of GOS and 2′-FL (mix 1 and mix 2), in some approaches were analyzed together as one condition (mixtures), and in others were analyzed separately.

### 2.7. Microbiota and Metabolite Analysis of Baby M-SHIME^®^ Experiments

#### 2.7.1. Quantitative PCR (qPCR) Analysis of Bifidobacteria

Quantification of *Bifidobacterium* was carried out on baby M-SHIME^®^ samples. The PCR mixture (total volume of 15 µL) contained a 5 µL template (between 1–10 µg/µL DNA, corresponding to a 1:100 dilution of the original DNA extract) and 10µL QPCR SYBR Green ROX Mix (Westburg), forward (Bif243F: TCGCGTCYGGTGTGAAAG) and reverse primer (Bif242R: CCACATCCAGCRTCCAC). Samples were incubated in a StepOnePlus real-time PCR device (Applied Biosystems). Negative controls for each batch of samples included a template consisting of qPCR water, while each sample was analyzed in triplicate. The amplification program used was: 15 min at 95 °C, followed by 40 cycles of 1 min at 95 °C and 1 min at 58 °C, and finally 105 s at 72 °C. Samples were checked for correct peaks in the melt curve. The standard curves had an efficiency between 95–105%. Three technical replicates of the qPCR were performed on each timepoint.

#### 2.7.2. Degenerative Gradient Gel Electrophoresis (DGGE)

DGGE profiling was applied to determine group-specific changes within the Bifidobacteria community. As each line in the fingerprint approximately represents one bacterial OTU, comparison of DGGE profiles, generated on samples at different timepoints throughout the experiment, allows the evaluation of qualitative changes in this group. After DNA extraction and PCR (7′ 95 °C; 35 × (1′ 94 °C/ 1′ 62 °C/2′ 72 °C); 10′ 72 °C 50–65%, 8%, 16 h, 38 V, 60 °C) with the Bifidobacteria group-specific primers, BIF164f and BIF662r [107], DGGE separated the PCR products. Gels were run using a DCodeTM Universal Mutation Detection System (Bio-Rad, Hercules, CA, USA), and data analysis was carried out using GelCompar, version 6.6 (Applied Maths, Sint-Martens-Latem, Belgium). Pearson correlation and UMPG clustering were used to calculate dendrograms of DGGE profiles.

#### 2.7.3. Identification of the Three Distinct Bifidobacterium OTUs by DGGE

Identification of the bifidobacteria included the creation of a clone library of PCR amplified 16S rRNA fragments, subsequent sequence analysis, and homology searches. By using selective primers, amplicons of a specific 500 bp fragment of the 16S rRNA gene of all different bifidobacteria present in a specific sample were obtained. These were used to create the clone library in *E. coli*, using the TOPO^®^ TA Cloning^®^ Kit for Sequencing (Life Technologies).

Upon collection of the clones, an inserted DNA fragment in the plasmid was amplified by direct colony PCR. This fragment was then sequenced. The resulting DNA sequences were subsequently compared with available databases for identification. The final step of the analysis included the selection of a representative clone from each of the different bifidobacteria species, followed by DGGE fingerprinting of both a selection of original samples and the clones. As the specific position of a band on a DGGE gel is determined by its DNA sequence, the unknown bands were then present at the same position in the gels as one of the specific clones, allowing these bands to be linked with a specific species identity. The three bands showed the following similarities with the 16S rRNA genes of *Bifidobacterium* strains: (i) 99% (470/471 bp) *B. adolescentis* strain ATCC 15703 (NCBI ref. NR_074802.1), (ii) 100% (466/466 bp) with *B. longum* strain NCC2705 (NCBI ref. NR_074744.1), and (iii) 100% (466/466 bp) with *B. longum* subsp. *infantis* strain ATCC 15697 (NCBI ref. NR_043437.1).

#### 2.7.4. Analysis of Metabolites

Metabolites were quantified in the supernatants of the fecal fermentation samples. SCFA (acetate, butyrate, caproate, propionate, and valerate) and branched chain fatty acids (BCFA; isobutyrate, isocaproate, and isovalerate) were measured using GC-MS as previously described [108]. Briefly, SCFA were extracted from the samples with diethyl ether, after the addition of 2-methyl hexanoic acid as an internal standard. Extracts were analyzed using a GC-2014 gas chromatograph (Shimadzu, ‘s-Hertogenbosch, The Netherlands), equipped with a capillary fatty acid-free EC-1000 Econo-Cap column (dimensions: 25 mm, 0.53 mm, film thickness 1.2 mM; Alltech, Laarne, Belgium), a flame ionization detector, and a split injector. The injection volume was 1 mL and the temperature profile was set from 110 to 160 °C, with a temperature increase of 6 °C/min. The carrier gas was nitrogen and the temperatures of the injector and detector were 100 and 220 °C, respectively.

Ammonium was measured as described previously [92]. Briefly, using a FoodALYT D 5000 Steam Distillation Unit (Omnilab, Voor ‘t Labo, Belgium), ammonium in the sample was liberated as ammonia by the addition of an alkali (MgO). The released ammonia was distilled from the sample into a boric acid solution. The solution was back-titrated with 0.02 N HCl using a Titroline (Omnilab, Voor ‘t Labo, Belgium).

## 3. Results

### 3.1. Effect of (the Combined) GOS and 2′-FL on Infant and Toddler Microbiota Composition and Diversity

To gain knowledge about the effects of (the combined) GOS and 2′-FL on infant and toddler gut microbiota composition, microbiota profiling of in vitro fecal batch fermentations, with these oligosaccharides added in different final concentrations and ratios, was performed using 16S rRNA sequencing.

The differences in microbiota composition after the fermentations with the supplements, as compared to the control, are visualized in Figure S1. For infants, the PerMANOVA analysis of the distances in microbiota composition only showed significant effects for the GOS treatments (Figure S1). The PerMANOVA analysis further showed that all supplements resulted in significantly different microbiota compositions compared to the medium control for toddlers. When comparing the resulting microbiota compositions of the two doses per supplementation tested (2.5 and 10 g/L), mostly overlapping clusters occurred, meaning that higher dosages of the ingredients did not induce additional shifts in microbiota composition compared to low dosages. Only for GOS treatment was a significant difference observed between both concentrations (*p* = 0.026) in the infant group (Figure S2).

**Figure 1:**
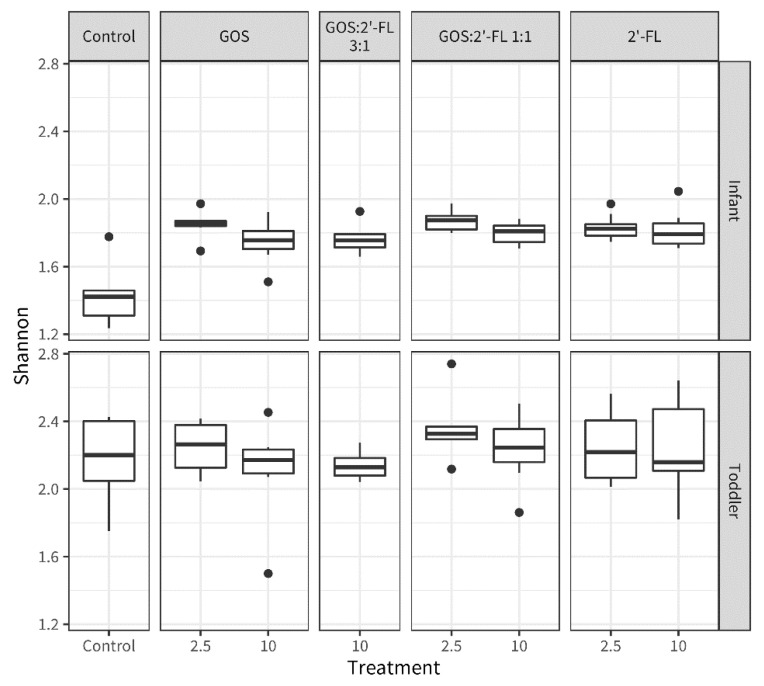
Boxplots representing the Shannon alpha diversity of the samples from the micro-Matrix bioreactor batch fermentations with fecal pools from infants (top) and toddlers (bottom) after 6 h of fermentation with GOS, 2′-FL, or mixtures thereof (GOS:2′-FL 3:1 and 1:1) at concentrations of 2.5 and 10 g/L, or without oligosaccharide (control). The boxes represent the interquartile ranges, thick vertical lines represent sample medians, the upper whisker ranges from the largest value within 1.5 × IQR (Interquartile range) from the hinge and the lower whisker ranges to the smallest value within 1.5 × IQR from the hinge.

The treatment effects were also reflected by an increase in microbiota alpha diversity in the supplemented samples compared to the medium control, on the basis of the Shannon index, in the infants but not the toddlers (Figure 1).

**Figure 2:**
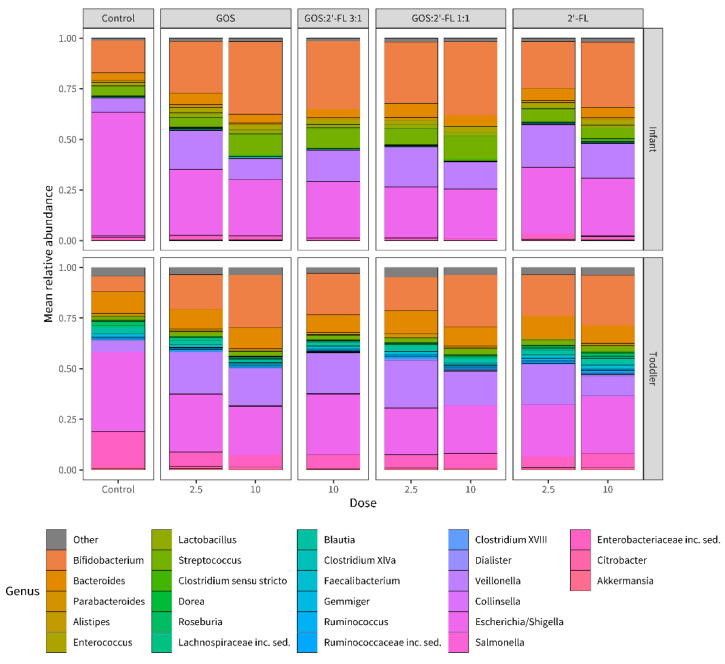
Relative abundance bar chart showing the top 26 abundant bacterial genera as determined by 16S rRNA gene amplicon sequencing. Results shown are from samples of the micro-Matrix bioreactor batch fermentation experiments after 6 h of fermentation with (GOS, 2′-FL, and mixtures thereof, as indicated in the figure), or without oligosaccharides (medium control), from the infant (upper bars) and toddler (lower bars) populations. The values on the x-axis represent the final concentration of oligosaccharides (2.5 and 10 g/L). The ‘other’ group includes bacterial genera accounting each for less than 1.79% and 3.79% of the reads for infant and toddler samples, respectively. Each bar represents the mean of the 6 replicates run for each condition.

The 26 genera with the highest relative abundances in the samples are visualized in Figure 2. It appeared that all the supplementations, at both concentrations, gave rise to comparable effects in fecal samples of both age groups after fermentation of 6 h. The relative abundances of *Bifidobacterium* and *Veillonella* were increased, while the relative abundance of *Escherichia/Shigella* was reduced, as compared to the control.

**Figure 3:**
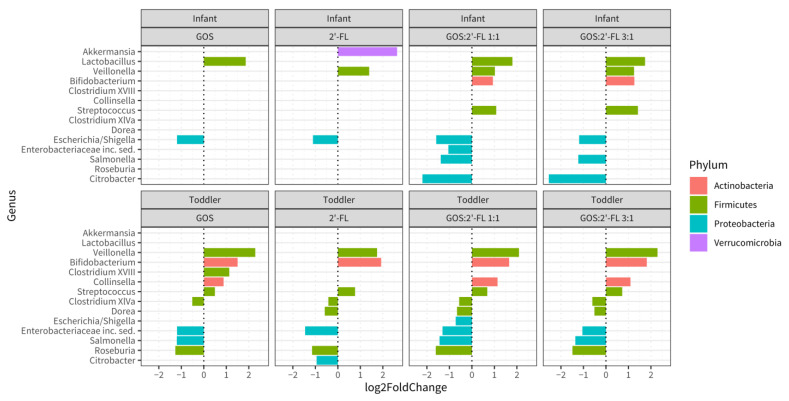
Barplots showing bacterial genera in samples of the micro-Matrix bioreactor batch fermentation experiments with significant differential relative abundance expressed as log2fold change between fermentations, with the indicated oligosaccharides and the control without oligosaccharides in the infant (**top**) and toddler (**bottom**) populations. Note that, outcomes from fermentations with the two dosages were compiled together and regarded as one treatment. Significance was tested by Wald test after fitting a negative binomial generalized linear model for each observed genus using the DESeq2 method.

To obtain insights into the significance of the observed differences, log2 fold changes for genera were determined for the supplemented fermentations (both concentrations compiled) compared to the control, and analyzed using the DESeq2 method (Figure 3). This revealed that, in the infant population, *Lactobacillus* was significantly increased by GOS, while *Veillonella* and *Akkermansia* were increased in 2′-FL fermentations. With both GOS and 2′-FL present, *Veillonella*, *Bifidobacterium*, *Lactobacillus*, and *Streptococcus* increased. *Escherichia/Shigella* were decreased in all supplemented samples as compared to the untreated ones, while *Salmonella* and *Citrobacter* were decreased only in samples from fermentations supplemented with both GOS and 2′-FL. The reduction in Proteobacteria was strongest for the combination of GOS and 2′-FL. Overall, in infants, the changes induced by the mixtures of GOS and 2′-FL were larger than those induced by GOS or 2′-FL alone.

In the toddler population, *Bifidobacterium* and *Veillonella* were increased in each of the supplemented samples after fermentation as compared to the control. Furthermore, *Clostridium*_XVIII was increased in the fermentations with GOS, whereas *Collinsella* and *Streptococcus* were increased in fermentations containing GOS and mixtures. *Citrobacter* was reduced only in fermentations with 2′-FL. In the presence of the mixtures and GOS, Salmonella was reduced, while 2′-FL and mixtures resulted in decreased *Dorea*. *Roseburia*, *Enterobacteriaceae*, and *Clostridium* XIVa were decreased in all supplemented samples compared to the control.

### 3.2. GOS, 2′-FL, and Mixtures Thereof Modulate the Infant Fecal Microbiota Composition and Activity in the Baby M-SHIME^®^ Gut Model

The dynamic baby M-SHIME^®^ model was employed to determine (sub)species specific bifidogenic effects by (combined) GOS and 2′-FL, and to determine the effects of (the combined) GOS and 2′-FL on the production of metabolites. The model was assembled with the part mimicking the proximal colon only, to allow the close comparison of five conditions in one run. The five parallel fermentations were repeatedly fed with the oligosaccharides in a final concentration of 3 g/L. The ratios of GOS and 2′-FL were: 100% GOS, 90% GOS and 10% 2′-FL (9:1), 75% GOS and 25% 2′-FL (3:1), and 50% GOS and 50% 2′-FL (1:1), and 100% 2′-FL.

**Figure 4:**
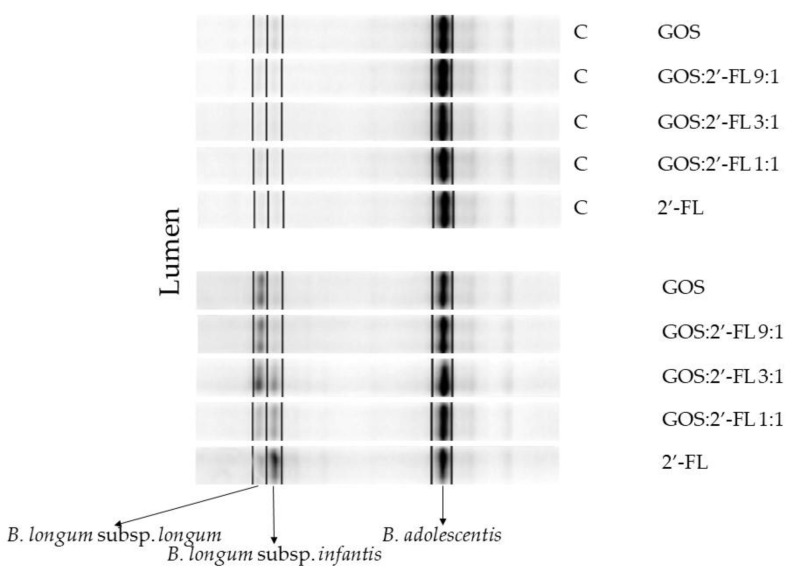
Pearson correlation of DGGE profiles of bifidobacteria in the lumen at the end of the control phase (C) and after 1 week of treatment with different combinations of GOS and 2′-FL, in the proximal colon of the baby M-SHIME^®^.

The generic bifidogenic effects of GOS and 2′-FL were similar in both fermentation models (data not shown). As the genus *Bifidobacterium* is composed of various infant-type and adult-type (sub)species, potential qualitative differences between the GOS and 2′-FL effects, with respect to the stimulation of specific *Bifidobacterium* species, were identified using DGGE (Figure 4). DGGE revealed the presence of one prominent identical *Bifidobacterium* OTU under all conditions, identified as *B. adolescentis*. Two other bands were present in samples of fermentations containing either GOS or 2′-FL. These bifidobacteria, that were differentially stimulated by GOS and 2′-FL, represent two subspecies of the species *B. longum*, namely *B. longum* subsp. *longum*, specifically stimulated by GOS, and *B. longum* subsp. *infantis*, specifically stimulated by 2′-FL. The same outcome was obtained with samples from the mucus compartment (Figure S3).

**Figure 5:**
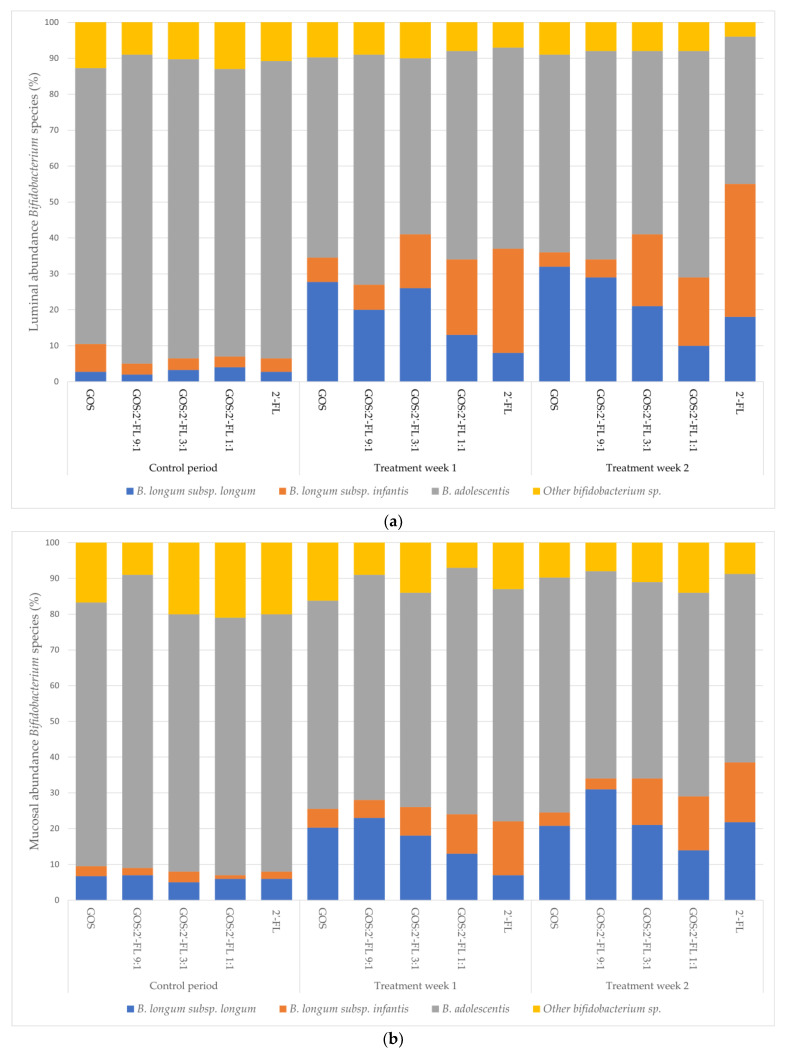
Relative share of three *Bifidobacterium* (sub)species in the luminal (**a**) and mucosal (**b**) microbiota in the proximal colon of the baby M-SHIME^®^ during the control and treatment phases (first week, TR1; second week, TR2), with different combinations of GOS and 2′-FL, as identified by DGGE.

A strong dose–response effect of increasing levels of 2′-FL on the stimulation of *B. longum* subsp. *infantis*, and of GOS on *B. longum* subsp. *longum*, could be discerned. Levels increased from 2–4% (*B. longum* subsp. *longum*) and 3–8% (*B. longum* subsp. *infantis*) in the control week, to 8–32% (*B. longum* subsp. *longum*) and 4–37% (*B. longum* subsp. *infantis*) of total bifidobacteria, with increasing dose during the two weeks of treatment (Figure 5). The species *B. adolescentis* was prominently present in all fermentations, but decreased from 77–86% in the control week to 64–49% during the two weeks of treatment. This seemed to happen in favor of the presence of the two *B*. *longum* subspecies.

Next, we assessed whether supplementation with GOS and/or 2′-FL also impacted the formation of metabolites in the infant fecal cultures. The typical fermentation products, SCFAs, lactate, BCFA, and ammonia were determined in the supernatants of fermentation samples. SCFA profiles were found to mainly consist of acetate, propionate, and butyrate, other acids were below the detection limit. Shortly after the introduction of the complex sugars, GOS, 2′-FL, and their mixtures significantly increased total SCFA levels in the proximal colon of the baby M-SHIME^®^ model, and continued to do so during the treatment, as shown in Table 1. A peak in lactate levels was also detected for the mixtures of GOS and 2′-FL.

Acetate, one of the metabolites produced by bifidobacteria, increased most strongly with the GOS and/or 2′-FL treatments. Acetate concentrations were significantly different from the control under all five conditions tested. Propionate and butyrate levels also increased during the treatment, albeit they were only partially significantly different from the control phase. In conclusion, GOS and 2′-FL, individually as well as together, enhanced SCFA production to a similar extent. The measured lactate, BCFA, and ammonia levels were not significantly different between GOS and 2′-FL fermentations compared to the control in this experiment.

## 4. Discussion

We employed two in vitro fecal fermentation models (batch and dynamic) to reveal the in vitro microbiota modulating effects of GOS, 2′-FL, and mixtures thereof, in infant- and toddler-derived fecal cultures. The experiments revealed that all treatments induced changes in microbiota composition, even at a low dose.

In the fecal fermentations, all supplementations resulted in an increased alpha diversity of the microbiota in the infant samples. This suggests that hMOs and GOS can support the shaping of the microbiota to more complex mature communities, that become more robust against perturbations and are able to process a great variation of dietary components reaching the colon. The different supplementations did not influence the Shannon index in the fecal samples derived from toddler fecal fermentations, probably because the baseline levels were already considerably higher in toddlers compared to infants, the latter being in accordance with earlier reports on increased microbiota diversity with age [109].

All supplementations showed rather similar microbial shifts for both age groups with respect to the 26 most abundant genera after fermentation in the micro-Matrix bioreactor batch cultures. This included an increase in the relative abundances of the genus *Bifidobacterium* compared to the control. This confirmed the well-established generic bifidogenic effect of GOS, as reported in vivo in infants [39,40,41,42,43,44], and was similar to previous in vitro studies on the microbiota modulating effects of GOS and 2′-FL [37,38,49,82,84].

DESeq2 analysis of the relative abundances of the 26 most abundant genera showed differential and synergistic effects for the single and combined prebiotics, respectively. Using this analysis, for infant samples, the bifidogenic effect was significant for the combination of GOS and 2′-FL in a ratio of 1:1 or 3:1, but not for the single components. However, for toddlers, in all fermentations a significant increase in the genus *Bifidobacterium* compared to the control was detected. This difference in outcome can probably be explained by differences in the initial levels of bifidobacteria, that were two times higher in the infant than the toddler samples at the start of the fermentations, leaving less room for improvement in the infants. In our study, the relative abundance of *Akkermansia* significantly increased in infant feces upon 2′-FL treatment, as was previously also demonstrated in mice [110,111]. *Akkermansia* has been shown to be able to use mucin as a carbon source [112]. Some of the terminal structures of mucin are similar to 2′-FL [113], which might explain the observed stimulation. Although GOS has also been shown to stimulate *Akkermansia* [114,115], no significant increase in *Akkermansia* was found in our experiments using GOS or mixtures of GOS and 2′-FL. For the mixtures, the absence of significant effects on *Akkermansia* could be explained by the lower concentration of 2′-FL as compared to the condition with solely 2′-FL.

*Lactobacillus* was also significantly increased in infant microbiota upon treatments including GOS in the batch cultures. Both genera, *Lactobacillus* and *Bifidobacterium*, are important producers of lactate and acetate [14,116,117,118], which are crucial for the decrease in luminal pH, Enterobacteriacea suppression, and as substrates for cross-feeding butyrate producers such as the *Clostridiales*, *Faecalibacterium prausnitzi*, or *Roseburia*. Butyrate, in turn, is amongst others known for its enterocyte “fueling” and anti-inflammatory properties [119,120,121]. With the increase in bifidobacteria and/or lactobacilli, Proteobacteria, including *Escherichia/Shigella*, *Salmonella*, and other genera belonging to the Enterobacteriacea family containing pathobionts, were suppressed by all supplementations in both age groups. The inverse relationship between the abundances of *Bifidobacterium* and proteobacteria in infant microbiota is in accordance with other studies [38,122,123,124]. Overall, the combination of 2′-FL and GOS (composed of many different structures in size and linkage type [96]) resulted in more complex microbiota shifts and more effective suppression of potential pathobionts, as compared to single oligosaccharides, in particular in infant feces.

In the M-SHIME model^®^ the microbiota composition shifts upon GOS and 2′-FL fermentation were accompanied by increased levels of SCFAs, in particular acetate. Early in the fermentations, lactate was increased as well, due to enhanced lactate production, putatively by *Streptococcus*, *Lactobacillus* and *Bifidobacterium*. The subsequent decrease in lactate levels during the experiment coincided with increased butyrate levels, possibly indicating cross-feeding between lactic acid producing bacteria and butyrate producing bacteria that use lactate as a substrate. As also *Veillonella* might profit from cross-feeding on lactate [125], this could possibly explain the increased relative abundance of *Veillonella* upon all treatments in toddler microbiota, as well as by treatments including 2′-FL in the infant microbiota in the batch fermentations. Enhanced SCFA production, reflecting saccharolytic fermentation, and decreased BCFA production, reflecting lower proteolytic fermentation, are generally regarded as beneficial [126]. Especially, acetate is known for its pH lowering and antimicrobial activity [15,16], and is the dominant SCFA in the infant intestine [14,46]. Changes in BCFAs and ammonia did not reach significance in the present experiment (data not shown). As proteolytic fermentation is believed to occur mainly in the distal colon, the lack of this part in the specific model setup employed in this experiment, could possibly underlie this outcome.

By applying DGGE, we observed differential stimulation of the two subspecies, *B. longum* subsp. *longum* and *B. longum* subsp. *infantis*, by GOS and 2′-FL, respectively, in the dynamic baby M-SHIME^®^ model. Both *Bifidobacterium* subspecies are important bacteria in the colon of breast-fed infants [127]. Although other studies mainly focused on the stimulation of *B. longum* subsp. *infantis*, known to thrive perfectly on fucosylated hMOs such as 2′-FL [75,78,79,80,128,129,130,131], *B. longum* subsp. *longum* strains are known to utilize hMOs as well [132,133]. Both subspecies were also shown to be stimulated by GOS in single strain growth experiments [35,134,135,136] and adult fecal batch culture [137]. To our knowledge, differential dose-dependent *Bifidobacterium* stimulating effects on the subspecies level in an infant fecal microbiota community background have not yet been reported. The ability to stimulate specific bacterial (sub)species is important with respect to the different beneficial properties of commensals, which are known to be (sub)species- and even strain-specific, rather than genera-specific. Besides the SCFA-mediated general benefits of bifidobacteria, strain-specific features, such as antimicrobial activity, including peptide secretion, virulence attenuation, anti-inflammatory activity, and protection against mucus deterioration, have been described [110,138,139,140,141,142,143,144]. Increasing the intra-genus diversity of breast-fed associated bifidobacteria, therefore, might be beneficial due to the potential increase in different beneficial features in the community. In addition, it has been demonstrated that feces from breast-fed infants harbored more different *Bifidobacterium* species in comparison to formula-fed infants [145]. In this respect, the effect of the combined application of GOS and 2′-FL could come closer to that of breast milk as compared to only one of these complex sugars.

The symbiotic relationship of both *B. longum* subsp. *infantis* and *B. longum* subsp. *longum* with their infant host is acknowledged [146]. While *B. longum* subsp. *infantis* is capable of utilizing any hMO structure [129], it has a preference for smaller molecules [75], *B. longum* subsp. *longum* strains are supposed to have the capability to use more complex oligosaccharides, including mucosal glycans [128,133,146,147]. This might explain the differential stimulation by the trisaccharide 2′-FL and the more complex GOS.

## 5. Conclusions

This study shows that GOS and 2′-FL, separately and combined, impact fecal microbiota composition, diversity, and activity in infants and young toddlers, in vitro. The relative abundance of bifidobacteria increased, and that of proteobacteria decreased, in response to both oligosaccharides (and their mixtures). This effect was already observed at a low dose of 2.5 g/L, but the increase in bifidobacteria was not significant for infant fermentations with single components. The infant microbial community showed more pronounced alpha diversity changes compared to the toddler community, although the shifts in microbiota composition were larger in the toddler community. The combination of 2′-FL and GOS resulted in more complex microbiota shifts and more effective suppression of potential pathobionts, as compared to single oligosaccharides, in particular in infant feces. The observed differential dose-dependent stimulation of the two *B. longum* subspecies, *longum* and *infantis*, by respectively GOS and 2′-FL, points to further potential benefits of the combination of GOS with 2′-FL, as both subspecies are key members of the healthy breast-fed infant microbiota.

## Figures and Tables

**Table 1:** Levels (mM) of acetate, propionate, butyrate, and total SCFA formed during the control (C) and treatment periods (first week, TR1; second week, TR2), with either 100% GOS or 100% 2′-FL, and different combinations of GOS and 2′-FL (depicted as 90/10, 75/25, 50/50 in percentages), in the simulated proximal colon of the baby M-SHIME^®^. The average amounts calculated from three samples per week are presented and significantly different values (*t*-test) are marked with an asterix.

Metabolite (mM)	Timepoint	GOS	GOS/2′-FL 9:1	GOS/2′-FL 3:1	GOS/2′-FL 1:1	2′-FL
Acetate	C	26.8	24.5	23.9	21.4	24.0
	TR1	35.7 *	33.9 *	32.4 *	29.4 *	33.6 *
	TR2	34.5 *	32.7 *	33.4 *	31.3 *	33.8 *
Propionate	C	7.7	6.5	7.2	7.0	7.2
	TR1	9.1 *	7.2 *	7.8 *	7.9 *	7.8 *
	TR2	7.9	7.1 *	7.2	7.2	7.2
Butyrate	C	8.8	8.9	8.6	9.5	8.2
	TR1	10.8 *	10.8	10.6	12.1	9.6
	TR2	11.5 *	11.5 *	11.0 *	11.2 *	10.6 *
Total SCFA	C	44.7	40.7	40.5	38.5	40.4
	TR1	57.4 *	53.3 *	51.7 *	49.9 *	52.4 *
	TR2	54.8 *	52.1 *	52.3 *	50.2 *	54.2 *

## Data Availability

The data supporting the findings of this study are available from the corresponding author [E.L] upon reasonable request.

## Supplementary Materials

The following supporting information can be downloaded at: https://www.mdpi.com/article/10.3390/children10030430/s1, Figure S1: Diversity analysis of microbiota composition; Figure S2: Influence of prebiotic concentration on microbiota composition; Figure S3: Bifidobacterial DGGE profiles of the mucosal microbiota.
