# Supplementary material for: Infant Fecal Fermentations with Galacto-Oligosaccharides and 2′-Fucosyllactose Show Differential Bifidobacterium longum Stimulation at Subspecies Level
Source: Children (Basel). 2023 Feb 23;10(3):430. doi: 10.3390/children10030430 (PMC10047592; doi:10.3390/children10030430)
Supplement: Supplementary file 1 [file children-10-00430-s001.zip › children-2100372-supplementary.pdf]

These supplementary data are part of the paper “Infant Fecal Fermentations with Galacto-Oligosaccharides and 2′-Fucosyllactose Show Differential *Bifidobacterium longum* Stimulation at Subspecies Level” by Lindner et al.

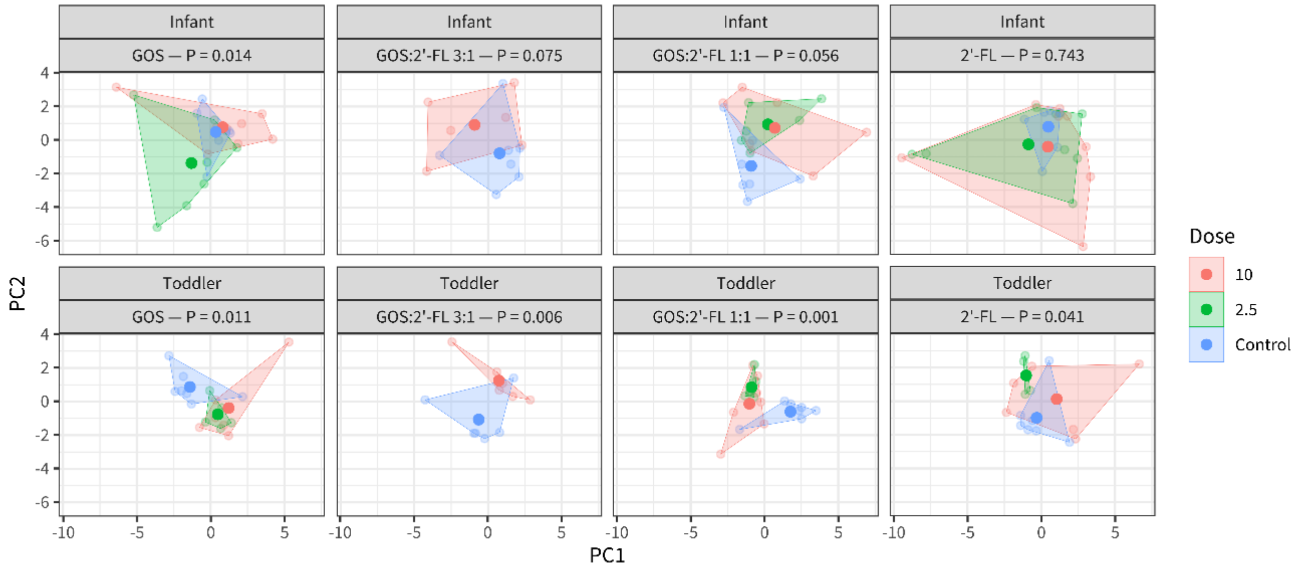

Figure S1. Diversity analysis of microbiota composition. Visual representation of the distances between the microbiota compositions of the fermentation samples from the two different doses (2.5 and 10 g/L) of the treatments with GOS, 2′-FL, and mixes and the medium control samples (control) for the infant (top) and toddler population (bottom). Note that *p*-values in the figures refer to the overall differences between all the groups represented in each figure and are not indicative of pairwise comparisons.

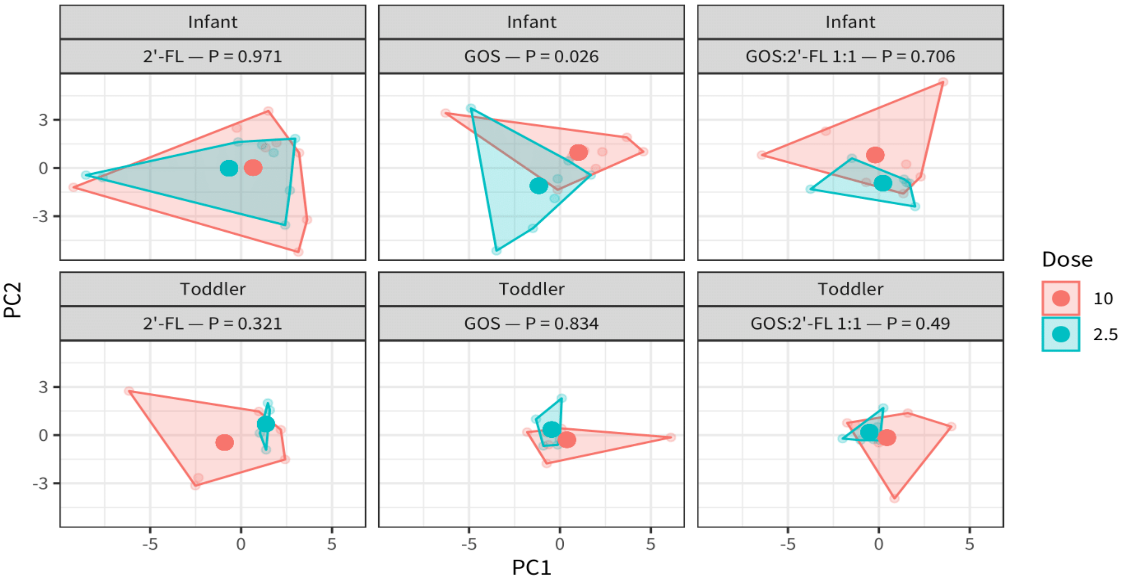

Figure S2. Influence of prebiotic concentration on microbiota composition. Visual representation of the distances between the microbiota compositions of the fermentation samples from the two different doses (2.5 and 10 g/L) of the treatments with GOS, 2′-FL, and mixes compared among each other for the infant (top) and toddler population (bottom).

Note that *p*-values in the figures refer to the overall differences between all the groups represented in each figure and are not indicative of pairwise comparisons.

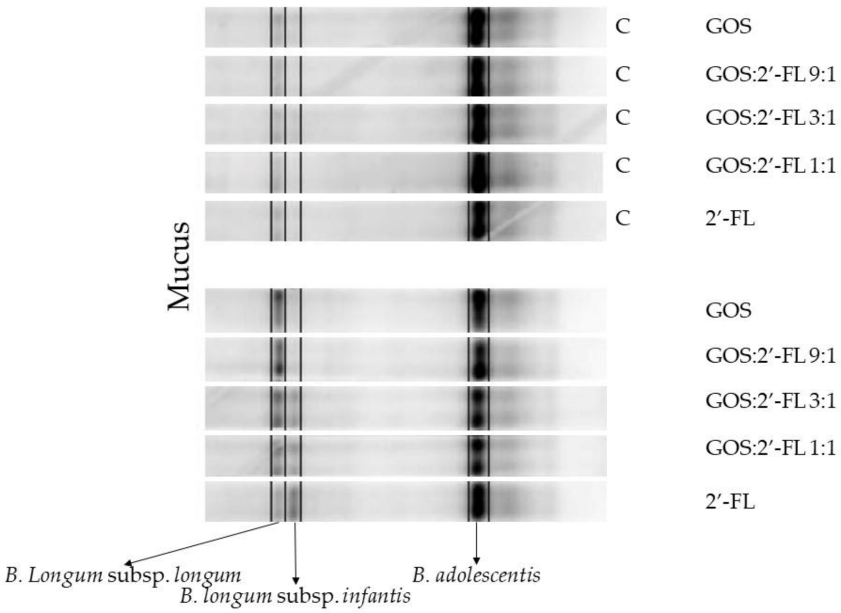

Figure S3. Bifidobacterial DGGE profiles of the mucosal microbiota. Pearsson correlation of the bifidobacterial DGGE profiles of the mucosal microbiota during the control and treatment period with different combinations of GOS and 2′-FL (depicted as 100/0, 90/10, 75/25, 50/50 and 0/100 of GOS/FL in percentages), in the proximal colon of the baby M-SHIME® in treatment week 1 and 2 (depicted as TR1, TR2) (n per week = 1).
